# Photodegradation of aqueous tetracycline using CuS@TiO₂ composite under solar-simulated light: Complete mineralization, catalyst efficiency, and reusability

**DOI:** 10.1016/j.heliyon.2025.e41662

**Published:** 2025-01-07

**Authors:** Ahed H. Zyoud

**Affiliations:** a Department of Chemistry, An-Najah National University, Nablus, Palestine; b Center of Excellence in Materials Science and Nanotechnology (CEMSANT), An-Najah National University, Nablus, Palestine

**Keywords:** Photodegradation, Photocatalyst, CuS/TiO₂, Tetracycline, Sensitization

## Abstract

While CuS/TiO₂ has been previously synthesized and employed in a limited number of photodegradation studies, the current study investigated its effectiveness for TC degradation under UV–visible light irradiation. CuS is known to be a nontoxic, environmentally friendly material; hence, it has great potential as an alternative to CdS and CdSe, which are used conventionally as sensitizers. In this work, the CuS/TiO₂ photocatalysts achieved a maximum 95 % removal of TC at an initial concentration of 20 ppm, confirming the good utilization of active sites. Even though the efficiency decreased for higher TC concentrations due to the saturation of the active sites, the values of the quantum yield showed that photon utilization was still effective. Consequently, the photocatalyst showed an optimum yield at 0.20 g, and its further addition increased the efficiency rather insignificantly. In addition to the near-complete mineralization of TC by the CuS/TiO₂ composite with few byproducts, its reusability was excellent because it showed almost consistent performance in successive cycles. These results further confirm the continuous relevance and potential of CuS/TiO₂ as a practical, sustainable solution for organic pollutant degradation, reinforcing its value in environmental remediation applications.

## 1 Introduction

Water pollution is currently one of the most serious environmental problems and poses a basic threat to ecosystem health and human well-being [[1], [2], [3]]. As sources of pollutants, industrial processes, agricultural runoff, and domestic wastewater introduce complex mixtures of organic and inorganic contaminants into aquatic systems [[4], [5], [6]]. These factors not only deteriorate water quality or pose major threats to wildlife and human populations but are also related to high social and economic costs as a result of the degradation of aquatic ecosystems [7,8]. These are quite diverse and persistent pollutants, whereas the conventional treatment techniques of filtration [9], chemical precipitation [10,11], and biological processes often fail to address this situation [12,13]. More advanced treatment technologies that can overcome complex contaminants are thus urgently needed.

Advanced oxidation processes (AOPs) have evolved into prominent tools for water treatment because of their ability to generate very reactive, high-activity species that can degrade a wide group of organic pollutants [[14], [15], [16], [17]]. Among these techniques, photocatalysis has gained wide consideration because of the potential of solar energy for use in driving the oxidation process; hence, it is very promising for achieving a sustainable concept in water treatment [[17], [18], [19], [20]]. Photocatalysis is the process by which semiconductor materials, upon illumination with light, generate reactive species such as the hydroxyl radical •OH and the superoxide anion •O_2_^−^. These radicals are highly efficient in the decomposition of organic pollutants into simpler compounds, which are much less harmful.

One of the most frequently used semiconductors in photocatalysis is titanium dioxide (TiO₂) because of its stability, nontoxicity, and strong oxidative ability [21,22]. Under ultraviolet light irradiation, TiO₂ normally generates electron–hole pairs, which are involved in redox reactions to generate active species for the degradation of pollutants as follows [23,24]:

TiO₂(VB) + hν (UV) → TiO₂(CB) + e^−^/h^+^

H_2_O + h^+^ → •OH + H^+^

O_2_ + e^−^ → •O_2_^−^

Although it is powerful under UV light, the photocatalytic activity of TiO₂ is limited to this slim part of the solar spectrum, covering only a minor fraction of sunlight. This greatly reduces the applicability of TiO₂-based photocatalysis in nature under sunlight conditions; therefore, developing strategies that can extend the operational spectrum is of prime importance. Sensitization is one such approach. The addition of other substances, called sensitizers, can enhance the visible light absorption ability of TiO₂, thus finally resulting in an extension of its photocatalytic activities from the UV region [25]. Cadmium sulfide and cadmium selenide [26,27], among others, have been traditionally used as sensitizers to sensitize the visible light response of TiO₂ [[28], [29], [30], [31]]. While these substances are quite efficient at increasing the photocatalytic activity in the visible region, their toxicity poses a great environmental and health risk [29]. Cadmium is a highly dangerous heavy metal that is nephrotoxic, osteotoxic, and likely carcinogenic in humans. In addition, cadmium compounds are potentially environmentally hazardous because of their ability to bioaccumulate and persist in ecosystems.

In this context, interest in finding substitutes that may help overcome the problems associated with the toxicity of Cd-based sensitizers is increasing. Among them, copper sulfide has recently been regarded as a potential alternative with reduced toxicity and more desirable optical performance. CuS is less toxic than cadmium-based materials and possesses an appropriate bandgap, which can cover visible light [32,33]. CuS in a TiO₂ matrix can extend the photocatalytic activity of TiO₂ to visible light, therefore enhancing its overall performance in photocatalytic degradation [34,35]:

CuS(VB) + hν (visible) → CuS(CB) + e^−^/h^+^

In the mechanism of CuS sensitization, visible light is absorbed by CuS, which increases the number of electrons to an excited state of CuS and results in their transfer to TiO₂(CB), leaving positive holes in the CuS(VB) [35]. This allows the material to be excited by visible light and enhances its photocatalytic efficiency. Thecoupling of CuS with TiO₂ not only resolves the issues related to conventional sensitizers but also fulfills the criteria of green chemistry in relation to environmental sustainability and a reduction in health risks.

**Scheme 1:**
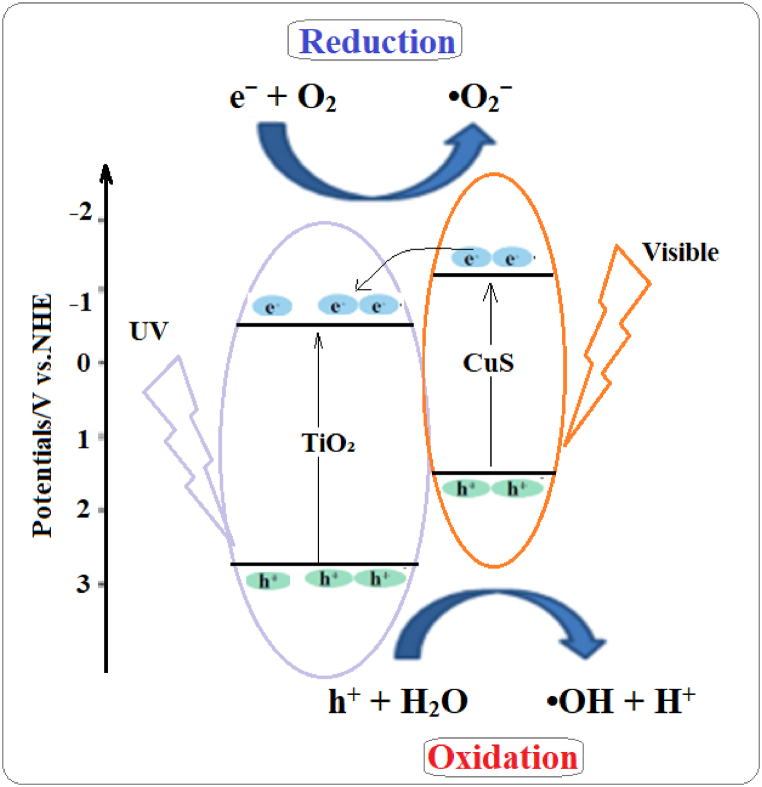
Schematic diagram of the sensitization of CuS to TiO₂.

The development of CuS/TiO₂ photocatalysts is an enormous improvement within the domain of photocatalysis. This method admirably couples TiO₂ stability and oxidation power with the visible light absorption capability of CuS, resulting in improved photocatalytic performance. Research on the use of CuS as a sensitizer aims for more efficient photocatalytic degradation while simultaneously trying to minimize the environmental and health impacts of hazardous materials (Scheme 1). The present research is related to the degradation of tetracycline (TC), a persistent pharmaceutical contaminant in wastewater, by CuS/TiO₂ systems [36,37]. TC is a substance that causes a myriad of serious environmental and health concerns because of its contribution to antibiotic resistance and persistence against conventional treatment methods [38,39].

This study focused on assessing the photocatalytic performance of CuS/TiO₂ under simulated conditions to resemble solar light. Metrics of the amount of TC removed, the percentage of TC removed, the turnover number (TON), and the quantum yield (QY) were evaluated in this study. Furthermore, the potential of the system for the complete mineralization of TC and its reusability by successive cycles will also be studied in this work. The obtained results will help in the development of safer and more effective technologies for wastewater treatment via photocatalysis, eliminate the shortcomings of classic sensitizers, and meet the requirements for environmentally friendly solutions.

While CuS/TiO₂ has been previously utilized in the photodegradation of some organic contaminants, its application has been limited. This study represents the first exploration of its use in the degradation of tetracycline, a persistent pharmaceutical pollutant. By optimizing the CuS/TiO₂ photocatalytic system specifically for this challenging pollutant, this research not only advances the material's application in photocatalysis but also supports the development of eco-friendly and efficient solutions for managing water pollutants. This approach lays the foundation for more effective and sustainable water treatment technologies.

## 2 Experiment

### 2.1 Materials and methods

Chemicals of high purity were used to obtain reliable and reproducible results. Copper sulfate (CuSO₄•5H₂O, 99.9 %) was supplied by Aldrich and used as a source of Cu^2^⁺ ions for the formation of copper sulfide (CuS) on the surface of the TiO₂ nanoparticles. Thiourea, obtained from Riedel-de Haën, had a purity of 99.85 % and served as a source of the sulfur ions S^2^⁻ required for the synthesis of CuS in the composite catalyst. In the synthesis of CuS, tararic acid (99.9 %) sourced from Aldrich served as a complexing agent to stabilize it through coordination with metal ions. pH-adjusted hydrochloric acid and sodium hydroxide (NaOH, 99.7 %) were supplied by Riedel-de Haën. Rutile TiO₂ powder (99.97 %), with an average particle size of 100–200 nm, was obtained from Aldrich and used as the primary photocatalytic substrate because of its high stability and photocatalytic activity. The tetracycline used as the target pollutant in the degradation studies was kindly donated by Birzeit Pharmaceutical Co.

The instruments used for this work were selected on the basis of precision and reliability. The solid-state electronic absorption spectra of the CdS films were recorded on a Shimadzu UV-1601 spectrophotometer using glass/FTO substrates for baseline correction to obtain accurate spectral data. The XRD patterns were recorded on a Philips XRD XPERT PRO diffractometer, which is a high-precision and high-sensitivity instrument, with all measurements taken in the UAE under the same environment. The morphology of the synthesized films was investigated via a Jeol JSM-6700F scanning electron microscope (Jeol Ltd., Japan). SEM analyses were performed in the UAE to guarantee that all samples were homogeneous by preserving consistency in sample preparation and image acquisition conditions. Photodegradation experiments were carried out using a 250 W tungsten-halogen lamp because of its very broad spectral range and high stability, which is able to simulate solar light with an average intensity of 0.667 W/cm^2^. In such a setup, the light from the plasma discharge source was both uniform and stable for the photocatalytic degradation studies.

### 2.2 Preparation techniques for CuS and CuS/TiO₂ composites

Various preparation methodologies have been employed in the context of photocatalysis for CuS and CuS/TiO₂ composites. The nature of the preparation methodology used considerably influences the morphology, photocatalytic activity, and stability of the material in question. To compare these techniques clearly, Table 1 presents various preparation methods that are proposed to be used to outline their respective merits and limitations.

**Table 1:** Comparison of preparation techniques for CuS in CuS/TiO₂ composites.

Method	Description	Expected Morphology	Key Advantages	Limitations
**Chemical Bath Deposition** [37]	Controlled deposition in a solution with temperature and pH regulation.	Uniform particle distribution, potential for nanostructuring	Good control over particle size and distribution, scalable	May require extensive washing to remove impurities.
**Hydrothermal Synthesis** [40]	Reaction under high temperature and pressure in a sealed vessel.	Nanorods, nanosheets, or nanoparticles	Can produce highly crystalline materials, versatile	High energy consumption, requires specialized equipment
**Solvothermal Method** [41]	Similar to hydrothermal but uses organic solvents, often resulting in different morphologies.	Often produces well-defined shapes	Can enhance the crystallinity and morphology control	Potentially hazardous solvents, longer processing times
**Sol-Gel Process** [42,43]	Formation of a gel from sol with subsequent drying and heat treatment.	Homogeneous, often amorphous before calcination	Simple, can produce uniform coatings or particles	Requires careful control of gelation and drying conditions
**Chemical Vapor Deposition** [44,45]	Deposition of material from vapor phase onto a substrate.	High purity, controlled thickness	High-quality films and coatings	Expensive, complex setup, and typically not suitable for large-scale production

The chemical bath deposition technique was employed for the preparation of the CuS/TiO₂ composite in this work, as it allows all the basic parameters of the pH and temperature of the solution and the deposition rate to be controlled with high precision, which is very important for achieving the desired morphology and performance. In this process, the active material CuS is uniformly distributed over the inert support provided by TiO₂, which plays a key role in increasing the photocatalytic efficiency. This methodology provides scalability and control and, therefore, is practical for the production of nanostructured materials with a well-dispersed CuS layer on TiO₂ particles. This morphology enhances charge separation and transfer efficiency, thus enhancing the photocatalytic degradation of tetracycline under visible light.

The morphology anticipated by this methodology is the uniform coating of CuS over the TiO₂ nanoparticles, increasing the active surface area and optimizing the photocatalytic performance of the composite. The uniform dispersal of CuS facilitates effective light absorption and the generation of reactive species that are necessary for the degradation of organic pollutants such as tetracycline. This method provides good adhesion of the CuS nanoparticles onto the surface of TiO₂, hence resulting in enhanced stability and reusability during photocatalytic reactions.

A controlled chemical deposition technique was employed for the synthesis of the CuS/TiO₂ composite. The corresponding preparation of 50 g of rutile TiO₂ powder stirred in 100 mL of 0.1 M copper sulfate pentahydrate (CuSO₄•5H₂O) and 0.1 M tartaric acid solution in a 250 mL beaker was conducted accordingly [[46], [47], [48]]. The pH was adjusted to 8.5 by adding a dilute 0.1 M NaOH solution dropwise from a burette and monitored by a pH meter to the required pH value. It was then heated to 80 °C by using a double-walled glass jacketed container connected with a thermostat for efficient and accurate temperature control. Heating was maintained for 15 min to establish optimum reaction conditions.

To this mixture, 50 mL of 0.2 M thiourea was added dropwise over a period of 15 min, and during this time, the mixture was subjected to stirring with a magnetic stirrer for proper mixing. After the addition of thiourea, stirring was further conducted for 60 more minutes at a constant temperature of 80 °C during the reaction. The mixture was allowed to settle, and the supernatant was removed. The composite powder was then washed three times in 1 L of distilled water to remove the unreacted ions and subsequently dried by air with calcination at 150 °C for 1 h [[48], [49], [50]].

This is indeed the prepared CuS/TiO₂ composite, which was advanced for further characterization and photocatalytic evaluation. The photocatalytic activity of the CuS/TiO₂ composite was tested via the degradation of an aqueous solution of tetracycline under controlled light conditions. Runs with degradation were repeated several times to determine the reusability and efficiency of the composite in water treatment.

### 2.3 Photodegradation experiments

To ensure the accuracy and reliability of the results, all the photodegradation experiments were performed according to a standard procedure. In particular, each experiment required a 250 mL beaker with an area of 38.5 cm^2^ to be filled with 100 mL of tetracycline solution of a 50 ppm. A 0.1 g of the CuS/TiO₂ composite or naked TiO₂ was subsequently added. The mixture was continuously stirred under the simulated solar light provided by a tungsten-halogen lamp with an intensity of 0.667 W/cm^2^ during the 60-min process. According to the experimental needs, the light source was either used directly or filtered with a 400 nm cutoff filter to isolate visible light [25,51,52].

Over the course of each photodegradation run, aliquots of the liquid were sampled at various time intervals. These samples were stored in the dark to prevent further photodegradation and centrifuged to remove solid particles, and the residual concentration of total cholesterol (TC) was measured spectrophotometrically. This procedure allowed for a precise determination of the degradation efficiency over time.

To explore the CuS sensitization effect and the ability to extend the activity of UV-limited TiO₂ into the visible spectrum, we carried out experiments involving TC solutions (100 mL, 50 ppm) mixed with 0.1 g of rutile TiO₂. These solutions were then irradiated by direct sources of light for an hour. Another set of solutions was irradiated under visible light via a 400 nm cutoff filter. For comparison, experiments have been performed using 0.1 g of the CuS/TiO₂ composite in place of rutile TiO₂ under similar conditions [53].

Further experiments were performed to study the influence of pH on the process of photodegradation. TC solutions (100 mL, 50 ppm) were mixed with 0.1 g of the CuS/TiO₂ composite, and the pH values were adjusted to 3.4, 4.6, 6.1, 7.2, 8.4, and 9.7. These mixtures were directly exposed to light for 1 h, with periodic sampling for analyses of the degradation rate. The set of experiments conducted hereby makes it possible to assess the effect of pH on the photocatalytic performance of the composite.

To examine the effect of the TC concentration on the photodegradation process, 0.1 g of the prepared CuS/TiO₂ composite was mixed with 100 mL of TC at different concentrations, including 20, 30, 40, 50, 60, and 70 ppm. The mixture was exposed directly to light for 1 h and continuously stirred. This allowed us to study the effect of the initial TC concentration on the efficiency of the photodegradation process.

Another batch of solutions of TC (100 mL, 50 ppm) with different quantities of the CuS/TiO₂ composite (0.05, 0.1, 0.15, 0.2, and 0.25 g) was prepared to study the effect of the amount of the CuS/TiO₂ composite on the degradation rate of TC.

For comparison purposes, several metrics were calculated, including the amount of photodegraded TC, percent removal, turnover number (TON), defined as the total number of TC molecules degraded per active site of the catalyst, and turnover frequency (TOF), defined as the number of TC molecules degraded per active site per unit of time.

For the complete degradation and mineralization of tetracycline (TC) during the photodegradation process, three analytical techniques were used. UV–visible spectroscopy and high-performance liquid chromatography (HPLC) were employed to monitor changes in TC concentration over time. A 100 mL solution of 50 ppm TC mixed with 0.1 g of CuS/TiO₂ was irradiated for 90 min. The samples were periodically analyzed every 15 min using UV–visible spectroscopy and HPLC to track the reduction of TC. Total organic carbon (TOC) analysis was performed on the same 100 mL solution at specific time intervals (0, 60, and 90 min) of irradiation using a TOC-LCSH/CPH Shimadzu analyzer to measure the mineralization of TC. These combined analytical techniques allowed for a comprehensive assessment of the CuS/TiO₂ composite's ability to achieve complete mineralization of TC during the photodegradation process.

## 3 Results and dissection

### 3.1 CuS/TiO₂ composite photocatalyst characterization

The structural, optical, and morphological properties of CuS/TiO₂ were characterized via a variety of techniques. Characterization methods are important in establishing how the modification of TiO₂ with CuS influences the photocatalytic behavior of the obtained composite. These techniques range from UV–visible spectroscopy and X-ray diffraction to scanning electron microscopy, among other methods, and provide information about the electronic structure and surface morphology of the composite material.

**Fig. 1:**
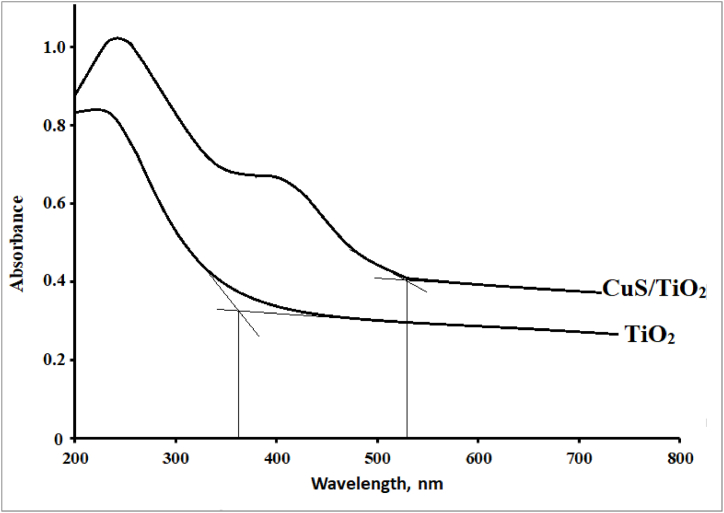
Solid-state UV–visible absorption spectra of naked TiO_2_ and the CuS/TiO_2_ photocatalysts.

In this section, the optical properties of the CuS/TiO₂ composite were studied via UV–visible spectroscopy. A small amount of the composite powder was dispersed in water and sonicated for 15 min to break up agglomerates into smaller, well-dispersed particles. The resulting dispersion, though not a homogeneous solution, ensured a uniform distribution of particles within the liquid. This prepared dispersion was then subjected to a UV–visible absorption scan in the wavelength range of 200–800 nm to analyze the optical absorption properties of the composite. The sonication process helped improve the reliability of the measurement by reducing particle agglomeration, thereby allowing for a more accurate assessment of the composite's optical characteristics [53]. As shown in the UV–visible absorption spectrum of the CuS/TiO₂ composite in Fig. 1, there are marked changes compared with those of pure TiO₂. The absorption spectrum of TiO₂ has an absorption edge at approximately 370 nm, corresponding to an energy of approximately 3.35 eV, thus limiting its photoactivity to the ultraviolet range. Modification by CuS results in the absorption edge of the CuS/TiO₂ composite being at 525 nm, with an energy of 2.36 eV, and hence extends the photoresponse into the visible light region. This shift in the absorption edge is considered strong evidence of CuS sensitization on TiO₂, hence exciting the composite system by both UV and visible light. The expanded absorption spectrum enhances the efficiency of the composite in regard to photocatalytic applications, as it increases its ability to utilize a wide portion of the solar spectrum for photodegradation applications.

**Fig. 2:**
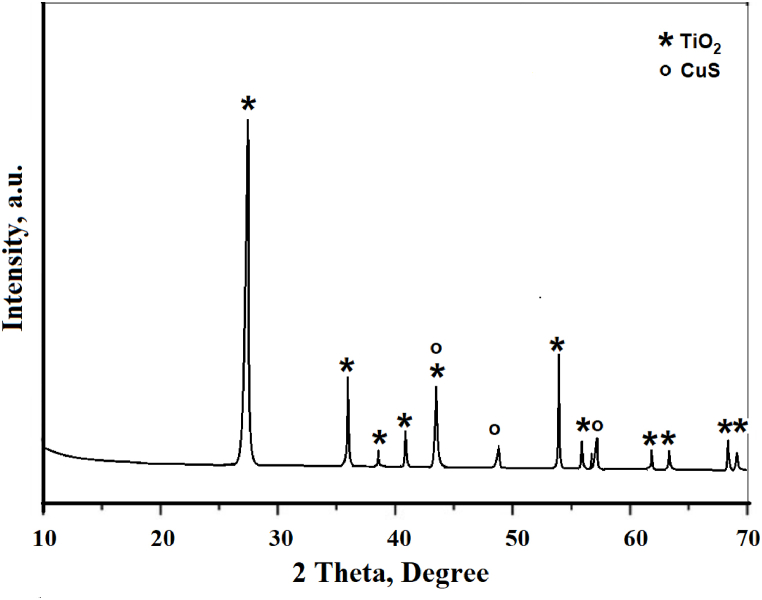
XRD pattern of the prepared CuS/TiO_2_ photocatalyst.

X-ray diffraction (XRD) was primarily performed to elucidate the crystalline structure of the composite material. Detailed information obtained from the XRD patterns of the phase composition and crystallite size confirmed the successful deposition of CuS on the TiO₂ surface. The diffraction peaks for TiO₂ and CuS confirmed the formation of a composite structure. The data obtained from the XRD analysis confirmed that the composite was very important for achieving structural integrity and phase purity to obtain efficient photocatalytic performance. The diffraction pattern of the CuS/TiO₂ composite powder is shown in Fig. 2.

The XDR patterns reveal the presence of rutile TiO₂ with diffraction related to the rutile TiO₂ card (JCPDS Card No. 21–1276) and reflections of CuS based on the card (JCPDS Card No. 06–0464). Calculations related to the Scherrer equation for particle size by reflection patterns for TiO₂ gave an average size of approximately 150 nm, whereas the calculations for reflections related to CuS provided an average particle size of approximately 15 nm.

**Fig. 3:**
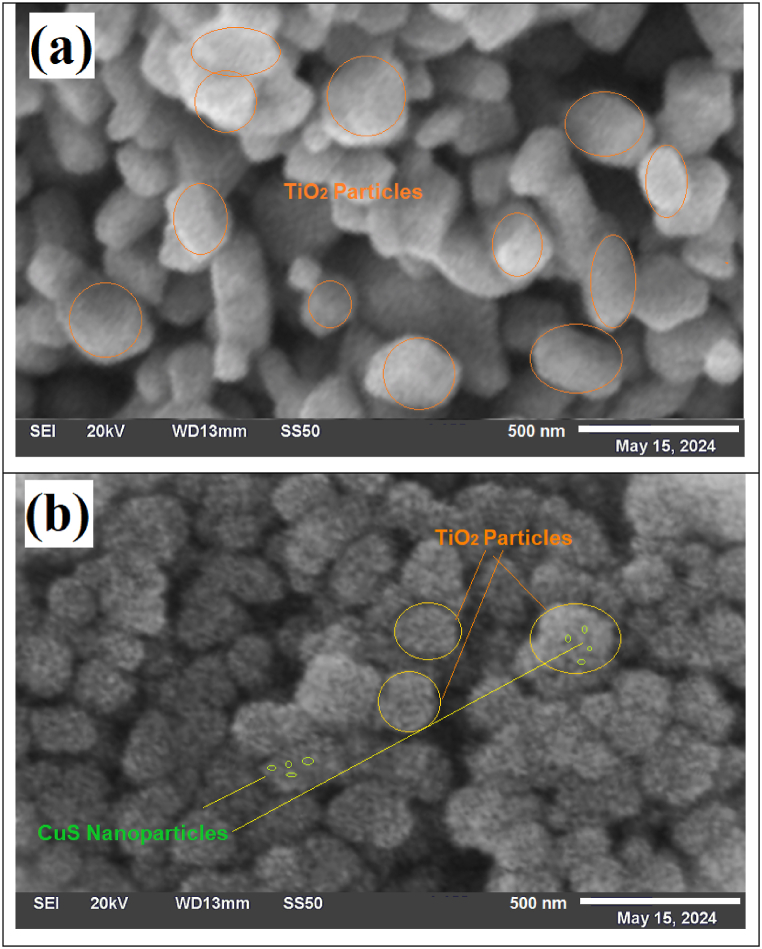
Scanning electron micrographs of a) the naked TiO_2_ powder and b) the CuS/TiO_2_ composite powder photocatalysts.

SEM was performed to study the surface morphology and particle size distribution of the synthesized composite CuS/TiO₂. For comparison, one more SEM image of naked TiO₂ was taken at the same resolution. Figure (3) shows SEM images of both naked and functionalized composites of TiO₂ with their nanostructure details.

The naked TiO₂ SEM image presented in Fig. 3a displays well-defined particles 100–200 nm in size, which is consistent with the expected morphology of the rutile form of TiO₂. These particles have a relatively smooth surface and do not display the added features seen in the composite and provide a basis against which the picture can be compared.

In contrast, for the CuS/TiO₂ composite presented in Fig. 3b, the CuS nanoparticles are dispersed well, with an average size of 15 nm, and are effectively anchored onto the surface of TiO₂. The finely dispersed CuS coating is one of the important factors that improves the photocatalytic performance of the composite, which can ensure good charge separation and increase the surface area for photocatalytic reactions.

A comparison of the two SEM images suggests that CuS has successfully attached to the surface of TiO₂, a key attribute for improving the photocatalytic properties of the composite. The fact that CuS nanoparticles are evenly coated on TiO₂, as in the case of the sensitization of CdS to TiO₂ reported earlier [53], provides assurance for the synergistic interactions between the two materials. This synergism is expected to be highly responsible for the improvement in photocatalytic activity, especially by extending the light absorption limit of the composite into the visible region.

Furthermore, SEM analysis confirmed that the morphology is not only homogeneous but also has an optimal particle size distribution, highlighting its potential for effective photocatalytic applications. The CuS/TiO₂ composite has a highly organized structure, as uniform dispersion of CuS contributes to enhanced photocatalytic performance, thus making this material promising for application in environmental remediation.

**Fig. 4:**
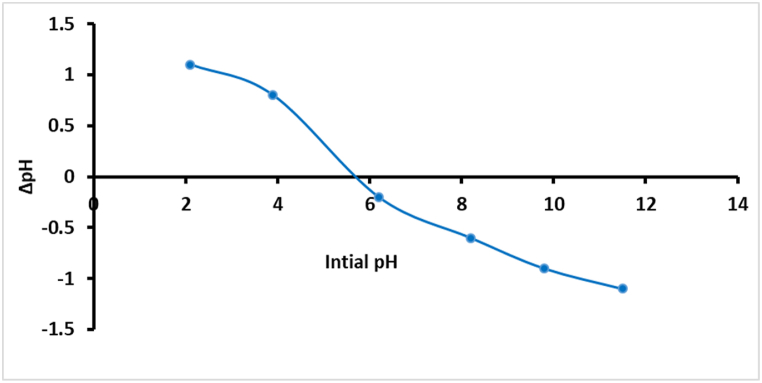
Plot of Δ(pH) vs. initial pH for the photocatalyst CuS/TiO_2_.

One of the critical parameters in assessing the surface charge properties of photocatalysts is the point of zero charge (pH_zcp_). This parameter strongly influences the interaction between photocatalysts and different pollutants involved in photodegradation processes. The pH_zcp_ is defined as the pH at which the net surface charge of the photocatalyst is zero. This parameter has a very significant influence on the adsorption of contaminants since the charge of the surface of the photocatalyst will affect its affinity for charged pollutants. The point of zero charge (pHzcp) of the CuS/TiO₂ composite was determined via the pH drift method in a 0.1 M KCl solution. Six 100 mL glass bottles, each containing 50 mL of 0.1 M KCl solution, were adjusted to initial pH values of 2.1, 3.9, 6.2, 8.2, 9.8, and 11.5. Subsequently, 0.1 g of the CuS/TiO₂ composite was added to each bottle. The capped bottles were then placed in a thermostated shaker at 25 °C and shaken for 24 h at 200 rpm. After the mixtures were allowed to settle, the final pH of each solution was measured. The relationship between the initial pH and the change in pH (ΔpH) is plotted in Figure (4).

Fig. 4 shows that the pH_zcp_ was approximately 5.7. More specifically, at pH values lower than 5.7, the surface of the composite catalyst will be positively charged, thus favoring the adsorption of negatively charged species, and inversely, it will be negatively charged at pH values higher than 5.7, thus promoting the adsorption of positively charged species. This means that determining the pH_zcp_ is one of the key steps toward the optimization of the photocatalytic reaction, as this provides insight into how the composite will interact with different pollutants at various pH values. This directly influences the effect of the charge behavior on the efficiency of contaminant adsorption and subsequent degradation, hence impacting the overall performance of the photocatalytic process.

The ratio of CuS to TiO₂ was determined by dissolving 0.01 g of CuS in concentrated HCl from the prepared photocatalyst. The resulting solution was analyzed for copper content via flame atomic absorption spectroscopy (FAAS), which revealed a CuS ratio of 1.2:100.

### 3.2 TC photodegradation results

The photodegradation experiments were conducted with 60 min of irradiation, following an initial 30-min dark stirring period to assess any potential TC loss due to adsorption onto the photocatalyst surface. No significant loss of TC by adsorption was observed during this period. The results, presented in the figures, show the photodegradation profiles, indicating the amount of TC remaining over time. The amount of photodegraded TC, percent removal, TON, and TOF metrics are summarized in Table 1 for the 30-min irradiation period.

**Fig. 5:**
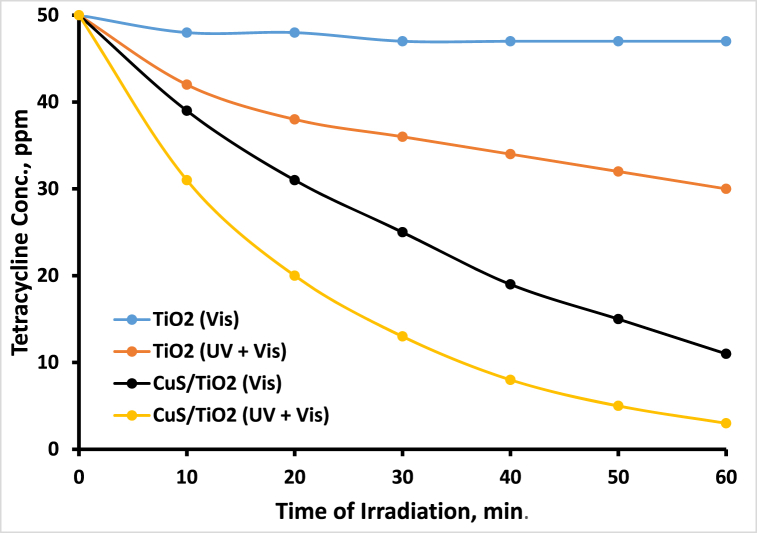
Photodegradation profiles of the different photocatalysts TiO_2_ and CuS/TiO_2_ under direct simulated light irradiation with a 400 nm cutoff filter.

Fig. 5 shows the photodegradation profiles of TC under TiO₂ and/or CuS/TiO₂ by direct irradiation (UV–visible) and by using a 400 nm filter. Under direct UV–visible irradiation, the TiO₂ photocatalyst achieved only 40 % degradation of tetracycline (TC). This limited efficiency is attributed primarily to the ability of TiO₂ to absorb only the UV fraction of the incident radiation, which is responsible for sensitizing the TiO₂. In contrast, when a 400 nm cutoff filter was used to block UV light, the photodegradation efficiency of TiO₂ decreased significantly, with only a 6 % reduction in TC observed. This minor loss is attributed to TC self-sensitization, wherein TC molecules adsorbed on the TiO₂ surface absorb visible light and become excited, acting as a dye to sensitize TiO₂ and thereby contributing to a modest increase in photodegradation activity.

On the other hand, the CuS/TiO₂ composite catalyst demonstrated superior photocatalytic performance, achieving up to 94 % degradation of TC under direct UV–visible irradiation within 60 min. When a 400 nm cutoff filter was employed, the composite still maintained a high photodegradation efficiency, achieving 78 % degradation of TC. This significant activity under visible light indicates the effective sensitization of TiO₂ by CuS, which primarily facilitates photodegradation through the absorption of visible light. The mechanism suggests that a substantial portion of the 94 % degradation is due to CuS absorbing visible light, whereas a smaller contribution comes from UV absorption by TiO₂. However, the presence of CuS can partially screen the TiO₂ surface, leading to a slight reduction in the efficiency of TiO₂ under UV light compared with the performance of pure TiO₂. This composite system thus highlights the enhanced photocatalytic properties of CuS in extending TiO₂ activity into the visible region, thereby improving the overall photodegradation efficiency compared with that of naked TiO₂.

**Fig. 6:**
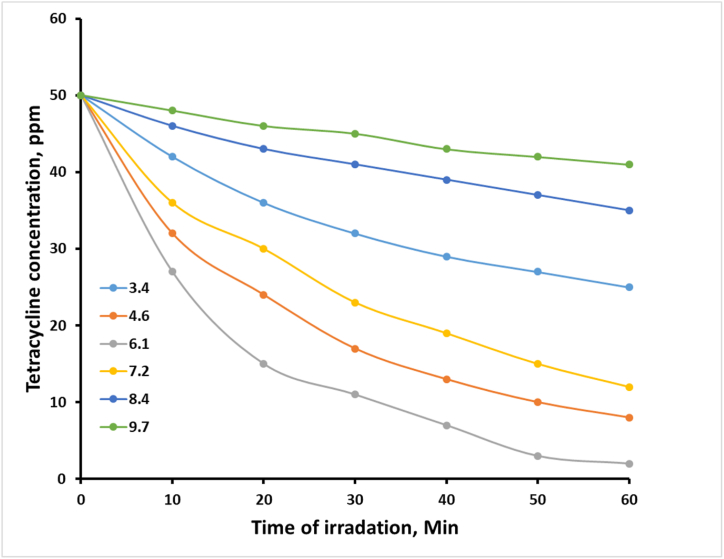
Photodegradation profiles of TC with time at different pH values.

In the photocatalytic degradation of tetracycline by CuS/TiO₂, the efficiency is intricately governed by the solution pH, surface charge of the photocatalyst, and speciation of TC. All these parameters have a profound influence on the adsorption behavior of TC over the surface of the photocatalyst and hence on the degradation efficiency. pH is also one of the critical factors that determines photocatalytic efficiency since it impacts the surface charge of the CuS/TiO₂ photocatalyst and the equilibrium TC structure. Fig. 6 shows that the zero charge point of the CuS/TiO₂ photocatalyst, pHzcp, is approximately 5.7. Below this pH, the surface of the photocatalyst is positively charged, whereas above this pH, it is negatively charged. These surface charge dynamics thus play a central role in dictating electrostatic interactions between the photocatalyst and TC molecules, which exist in different forms according to the pH conditions—for instance, in the ionic form.

At pH 3.4, the prevailing forms of TC molecules are cationic in nature, that is, TC⁺, with a considerable amount existing in the zwitterionic form, denoted by TC⁰. It is expected that at such an acidic pH, the positive charge on the photocatalyst surface is electrostatically repulsive with TC⁺, which may hinder adsorption. Nevertheless, the existence of nonelectrostatic forces—hydrogen bonding and van der Waals interactions—permits the adsorption of zwitterionic TC⁰. These interactions keep the TC within a close enough distance from the photocatalyst surface to support 56 % efficient photodegradation. Although modest, this efficiency exemplifies the intricate interplay of electrostatic repulsion and noncovalent attractions to enable TC adsorption and subsequent degradation.

At pH 4.6, TC is a mixture of cationic, TC⁺, and zwitterionic, TC⁰, species, with a slight predominance for the latter. Owing to their dipole moment, zwitterionic species interact more favorably with the positively charged photocatalyst surface. This in turn reduces the overall electrostatic repulsion and enhances the adsorption of TC onto the catalyst surface. Accordingly, the photodegradation efficiency increased to 66 %. The improved interaction could be ascribed to dipole‒dipole interactions and hydrogen bonding between the zwitterionic TC and the photocatalyst surface, which facilitates an improved degradation rate. A further increase in the pH to 6.1 resulted in nearly neutral conditions around the pH of the CuS/TiO₂ photocatalyst. Under these conditions, the photocatalyst surface is close to neutral; hence, electrostatic interactions are very minimal. At this particular pH, TC is primarily in its zwitterionic form (∼90 %), with an increasing share of anionic species in the form of TCˉ. Owing to the neutrality of the surface charge, optimum adsorption of the zwitterionic species of TC is allowed without considerable repulsive forces. This proximity to the photocatalyst active sites ensures maximum interaction with the generated hydroxyl radicals, thereby achieving the highest efficiency of photodegradation at 78 %. This finding also underlines the role of charge neutrality in maximizing adsorption and photocatalytic processes.

Finally, an increase in the pH to 7.2 results in a change in the equilibrium of TC, slowly biasing toward its anionic form, TC⁻. The negative charge on the photocatalyst surface may repel the anionic species of TC⁻, reducing the adsorption efficiency. Although some zwitterionic TCs still exist, an increased fraction of the anionic form may contribute to lowering the interaction with the surface, causing a reduction in the photodegradation efficiency to 54 %. Although it has already decreased, photodegradation is still enhanced because of the attraction between the positively charged dipole of the zwitterion, TC⁰, and the negatively charged photocatalyst. At pH 8.4, TC is mainly in the anionic form as TC⁻, with a minor presence of the dianionic form as TC^2^⁻. As a result, these anionic species are strongly repelled by the negatively charged surface of the photocatalyst, resulting in significantly reduced adsorption. Consequently, TC molecules are not appropriately positioned near the depletion layer of the photocatalyst, which is rich in hydroxyl radicals. This poor positioning results in a drastic decrease in the efficiency of photodegradation to 18 %. This large decrease indicates the negative impact of charge repulsion on adsorption and, therefore, on photocatalytic degradation.

At pH 9.7, TC mainly exists in its dianionic form, that is, TC^2^⁻. The surface of the photocatalyst remains negatively charged because severe electrostatic repulsion takes place, which allows a very minute amount of TC to adsorb on the surface of the catalyst. This strong repulsion prevents effective adsorption and proximity to the reactive hydroxyl radicals and is thus connected with the lowest photodegradation efficiency of 10 %. This dramatic drop clearly illustrates that, driven by favorable surface charge interactions, adsorption is a requirement for efficient photodegradation. In addition to electrostatic interactions, chemical interactions such as hydrogen bonding and van der Waals interactions are important contributors to the adsorption process. Hydrogen bonding, facilitated through the hydroxyl groups on the TiO₂ surface, is very effective at near-neutral to slightly acidic pH values, which favors the TC structure for such interactions. This type of chemical interaction complements the zwitterionic form of TC, thus efficiently adsorbing and therefore enhancing photodegradation efficiency. van der Waals forces provide ancillary support, especially when electrostatic interactions are not favored. Even at low pH, where TC is mostly in the cationic form, these forces may still contribute to the maintenance of some degree of adsorption against electrostatic repulsion. The sum of these chemical interactions ensures that the TC molecules are kept close enough to the photocatalyst active sites and make as many hydroxyl radicals as possible available for their degradation into less harmful byproducts.

The turnover number and quantum yield may provide insight into the process of photodegradation. The TON refers to the number of TC molecules degraded per active site on the photocatalyst; thus, the highest TON is observed at pH 6.1, which reflects the optimum utilization of the catalyst's active sites (Table (1)). The quantum yield is defined as the efficiency of the conversion of light absorbed into chemical reactions; therefore, it peaks at pH 6.1 when the catalyst works with the highest efficiency in providing light energy for degradation. This result is correlated with the optimal conditions for adsorption. In summary, the CuS/TiO₂ photocatalyst works efficiently in the photodegradation of tetracycline and strongly depends on the pH of the solution. The optimum conditions for maximum photodegradation were found to be pH 6.1, at which the surface charge of the photocatalyst balances that of the ionic form of TC to achieve efficient adsorption and proximity to hydroxyl radicals, hence affecting degradation. This relationship is important in optimizing photocatalytic processes for environmental applications.

### 3.3 Effect of TC concentration on the photodegradation rate

**Fig. 7:**
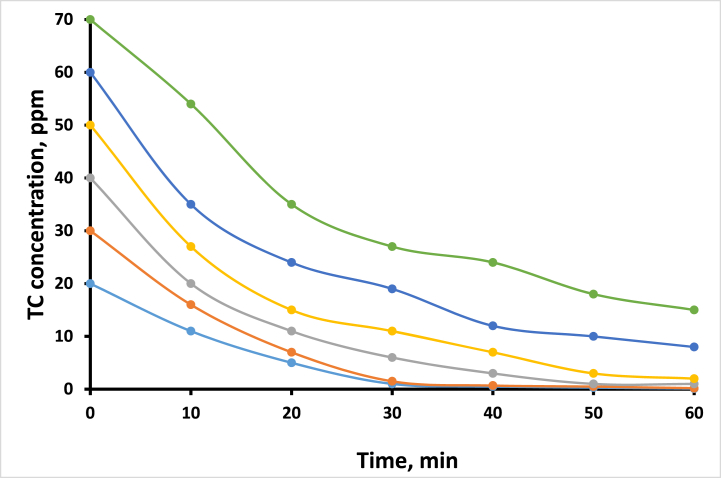
Photodegradation profiles of TC with time at different TC concentrations.

Among the parameters controlling the efficiency of photodegradation by the CuS/TiO₂ photocatalyst is the initial concentration of tetracycline (TC). This work focused on the degradation performance with different TC concentrations, such as 20, 30, 40, 50, 60, and 70 ppm, under simulated solar light for 30 min. A period of 30 min was chosen to indicate the degradation efficiency by concentration, as extended periods mostly result in nearly complete degradation at lower concentrations and can lead to confusing comparative analysis. The evaluations were based on three key parameters: % removal of TC, TON, and QY, as presented in Table 2. At the lowest concentration of 20 ppm, the photocatalytic system demonstrated a very high removal efficiency of 95 %, where 19 ppm TC was degraded (Fig. 7). This high efficiency is caused by the ample availability of active sites on the surface of the photocatalyst in relation to the number of TC molecules, allowing optimal adsorption and subsequent degradation. These QY and TON values reflect the efficient conversion of the absorbed photons into a chemical reaction, resulting in a robust photodegradation process. When the concentration increased to 30 ppm, the removal efficiency remained at 95 %, where 28.5 ppm TC was removed, which was consistent with the trend obtained at 20 ppm. This finding confirms that under such conditions, the CuS/TiO₂ photocatalyst is robust because there are still adequate active sites to catalyze high degradation efficiency.

**Table 2:** Removal efficiency parameters of the TC photocatalyst under various conditions.

**Effect of Type of Irradiation on TC Removal**				
Photcatalyst (Irradiation type)	Amount Removed (ppm)	% Removal	TON (x 10³)	QY
TiO₂ (Vis)	3	6 %	0.54	0.011
TiO₂ (UV–vis)	14	28 %	2.52	0.053
CuS/TiO₂ (Vis)	25	50 %	4.50	0.095
CuS/TiO₂(UV–Vis)	37	74 %	6.66	0.14

**Effect of pH on TC Removal**				
pH	Amount Removed (ppm)	% Removal	TON (x 10³)	QY

3.4	28	56 %	5.04	0.106
4.6	33	66 %	5.94	0.125
6.1	39	78 %	7.02	0.148
7.2	27	54 %	4.86	0.102
8.4	9	18 %	1.62	0.034
9.7	5	10 %	0.90	0.019

**Effect of TC Concentration on Removal**				
TC Conc. (ppm)	Amount Removed (ppm)	% Removal	TON (x 10³)	QY

20 ppm	19	95 %	3.42	0.072
30 ppm	28.5	95 %	5.13	0.108
40 ppm	34	85 %	6.12	0.129
50 ppm	39	78 %	7.02	0.148
60 ppm	41	68 %	7.38	0.155
70 ppm	43	61 %	7.74	0.163

**Effect of Photocatalyst Loading on Removal**				
Catalyst Amount (g)	Amount Removed (ppm)	% Removal	TON (x 10³)	QY

0.05 g	25	50 %	9.00	0.095
0.10 g	35	70 %	6.30	0.132
0.15 g	44	88 %	5.28	0.167
0.20 g	46	92 %	4.14	0.174
0.25 g	47	94 %	3.38	0.178

However, when the concentration of TC was increased to 40 ppm, the removal efficiency decreased to 85 %, with 34 ppm of TC degraded. This can be attributed to active site saturation, whereby the TC molecules start to outnumber available sites on the photocatalyst surface. Notably, at this concentration, the QY increased, meaning that while the proportion decreased, the absolute number of degraded TC molecules increased. This translates to effective photon utilization, where constant photon adsorption at high concentrations contributes to a high QY; hence, the ability of the photocatalyst to effectively convert the absorbed light into a chemical reaction under competitive conditions. At 50 ppm, it further decreased to 78 %, with 39 ppm TC removed, possibly indicating increased competition for limited active sites. The QY further increased, indicating that an increasing number of absorbed photons were being utilized effectively even when the photocatalyst's capacity was increased. This trend underlines the balance of photon efficiency against active site saturation, where it is obvious that the system has the ability to process more TC molecules despite its limited adsorption capacity. The degradation efficiency decreased at higher concentrations ranging from 60 ppm to 68 %, where 41 ppm TC was degraded, which was a critical threshold since severe active site saturation affects performance. Nevertheless, the QY tended to increase, indicating that the photocatalyst effectively transferred the absorbed photons into degradation reactions despite further optimization of the catalyst that may be required under high loads.

Finally, at the highest concentration tested (70 ppm), the removal efficiency decreased further to 61 %, in which 43 ppm TC was removed, emphasizing that a high TC concentration matters. Although the QY manifested continued photon efficiency, the reason behind the reduced effectiveness was due to an insufficient number of active sites holding onto such high degradation rates. This scenario is one in which structural optimization of a photocatalyst plays an important role in increasing the availability of active sites for improved performance at higher concentrations. The overall trend of the data clearly indicates that with increasing TC concentration, the photodegradation efficiency decreases, mainly due to the limited availability of active sites over the photocatalyst surface. At low concentrations, sufficient active sites are available to adsorb and degrade TC molecules efficiently, thus resulting in higher removal efficiencies. However, with increasing concentration, some form of competition intensifies, thereby decreasing adsorption and ultimately lowering the degradation efficiency. This underlines the optimization of photocatalyst loading and the need for enough active sites to achieve and sustain high performance with variations in pollutant concentrations. Moreover, this photocatalyst was able to effectively absorb light during the degradation process, as evidenced by the increase in QY with increasing TC concentration. The QY increases as the number of adsorbed photons remains constant, and the corresponding number of degraded TC molecules tends to increase. This efficiency shows that even under high-load conditions, in which active site saturation could further impair performance, the photocatalyst efficiently converts the absorbed photons into a chemical reaction.

### 3.4 Effect of the amount of loaded photocatalyst

**Fig. 8:**
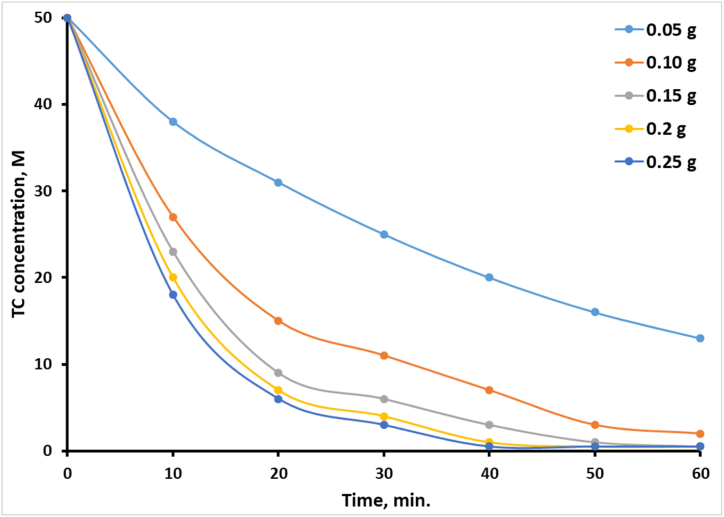
Photodegradation profiles of TC with time when different amounts of loaded photocatalyst were used.

Research on the effects of varying amounts of photocatalysts on TC photodegradation efficiency has revealed some vital facts about the influence of catalyst loading on the degradation process. During this research, various masses of the CuS/TiO₂ photocatalyst (0.05, 0.10, 0.15, 0.20, and 0.25 g) were used in a solution of 100 ml of 50 ppm TC under direct solar-simulated light irradiation for 30 min. The pH was adjusted to 6.1, which is optimal for photocatalytic activity. The photodegradation profiles of TC with different amounts of loaded photocatalyst over time are shown in Fig. 8, and the results, summarized in Table 2, outline trends in the amount of TC removed, percentage removal, TON, and QY with increasing amounts of photocatalyst.

At 0.05 g, the efficiency was 50 %, with 25 ppm TC removed. The low removal efficiency at this load reflects the limited number of active sites available for the adsorption and degradation of TC. When the amount of catalyst was increased to 0.10 g, the removal efficiency increased to 70 %, with 35 ppm of TC degraded, thus proving that with an increase in amount, more active sites are present for adsorption and degradation. The TON decreased with increasing catalyst mass, suggesting that the efficiency of using individual active sites decreases with increasing catalyst mass. However, the QY continued to rise, thus proving that the system's ability to translate the absorbed photons into chemical reactions improved with increasing catalyst loading.

The photocatalyst amount was then doubled to 0.15 g, but the removal efficiency increased to only 88 %, corresponding to 44 ppm of TC removed. Although the TON further decreased with decreasing efficiency per active site, this further increased the QY, reflecting more efficient photon-to-chemical conversion. By 0.20 g, the removal efficiency reached 92 %, with the QY still remaining very high. This means that as the photocatalyst approaches its maximum capacity, the photon efficiency remains robust. The removal efficiency was highest at 0.25 g, where it reached 94 % at 47 ppm TC. Despite the highest removal efficiency, the TON continued to decrease, indicating a decrease in the efficiency of the active sites, probably due to catalyst aggregation or reduced light penetration. The QY remained high, indicative of effective photon use even with the system approaching saturation.

This study has therefore underscored the need for an optimal amount of photocatalyst to achieve effective TC photodegradation. On the one hand, increasing the amount of photocatalyst increases the availability of active sites and hence the removal efficiency. On the other hand, there is a diminishing return in the TON with more catalyst since the individual site efficiency actually decreases. In contrast, the QY increases, indicating that the system becomes more effective at converting absorbed photons into degradation products with higher catalyst loading. These findings indicate that the availability of active sites balances with photon utilization, thereby establishing that the optimum catalyst loading falls within the range of 0.15–0.20 g. Beyond this optimum range, more catalysts do not improve the degradation efficiency, and in excess, they can create practical problems. In this context, optimization of catalyst loading is critical to maximize photodegradation rates and effectively utilize light energy in the treatment of wastewaters.

### 3.5 Complete mineralization of TC and reusability studies

**Fig. 9:**
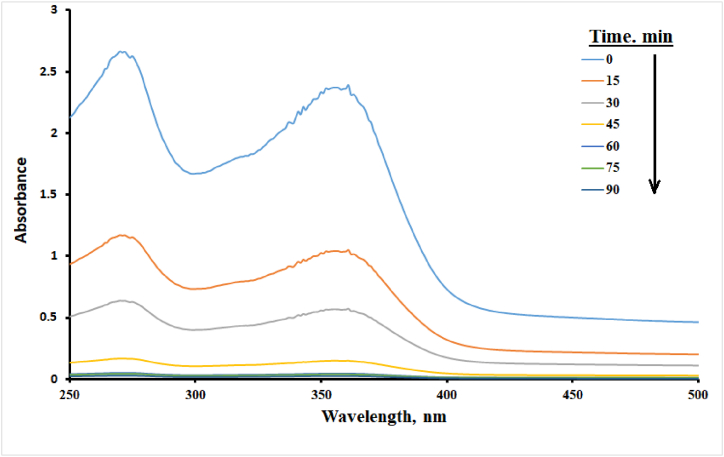
UV–visible absorption spectrum of the TC with time.

The complete mineralization of tetracycline (TC) in the photodegradation process assisted by the CuS/TiO₂ photocatalyst provides very significant information concerning the efficiency and effectiveness of this system for the treatment of organic pollutants. In this work, a solution containing 50 ppm TC in 100 mL was subjected to solar-simulated irradiation in the presence of 0.1 g CuS/TiO₂ for 90 min, with samples aspirated every 15 min for detailed analysis. UV–visible spectrophotometric analysis indicated that there was a significant decrease in the TC concentration with respect to time, as indicated by the gradual decrease in the absorption peaks characteristic of TC, as depicted in Fig. 9. The number of spectral peaks decreased, which indicated that photodegradation was effective and resulted in the breakdown of TC into compounds of less complexity. To further validate the extent of its mineralization, an HPLC‒DAD analysis was conducted using a C18 column. The mobile phase consisted of 0.01 % phosphoric acid and acetonitrile at a volume ratio of 2:1 and was eluted in isocratic elution mode. Initially, a peak for TC at t_R_ = 4.1 min was observed, which decreased with irradiation time until it had almost vanished by the end of the process, thus confirming the degradation of TC and indicating that it had been almost completely mineralized by the end of the irradiation. Other evidence of total mineralization was provided by the TOC analysis, which revealed that the TOC content decreased from 30 ppm in the freshly prepared 50 ppm TC solution to 12 ppm after 60 min and became undetectable after 90 min. This proves the efficacy of the photocatalyst in achieving total mineralization, in which TC is completely converted to nontoxic inorganic products with no stable organic intermediate compounds, which is very significant for effective environmental remediation.

Apart from the complete mineralization of carbon, considerable transformation of nitrogen occurred during the process of photodegradation. The nitrogen atoms in the TC were first ionized into less stable ammonium ions (NH_4_^+^), which subsequently became oxidized into more stable nitrate ions (NO_3_^−^). After 90 min of irradiation, approximately 6.5 ppm nitrate ions corresponding to 1.5 ppm nitrogen were detected, which was significantly lower than the theoretical yield of 3 ppm nitrogen expected for complete mineralization. This disparity may be due to the volatilization of nitrogen from the total nitrogen content into the atmosphere as diatomic nitrogen gas in the form of N_2_ or as NH_3_ by escaping ammonia gas; hence, a portion of the nitrogen content may have been released into the atmosphere. These nitrogen transformations provide deep insight into the complex dynamics of photocatalytic reactions and highlight the need to consider all possible nitrogen pathways, including gaseous emissions, to make a more accurate estimate of the overall environmental impact of photocatalytic treatment systems. The successful conversion of nitrogen compounds together with organic carbon highlights the enhanced ability of the photocatalyst to treat a far wider range of contaminants, thereby increasing its applicability for complete wastewater treatment. These insights underscore the ability of CuS/TiO₂ photocatalysts to successfully manage both organic and inorganic pollutants, thus offering a holistic solution to complex wastewater challenges.

**Fig. 10:**
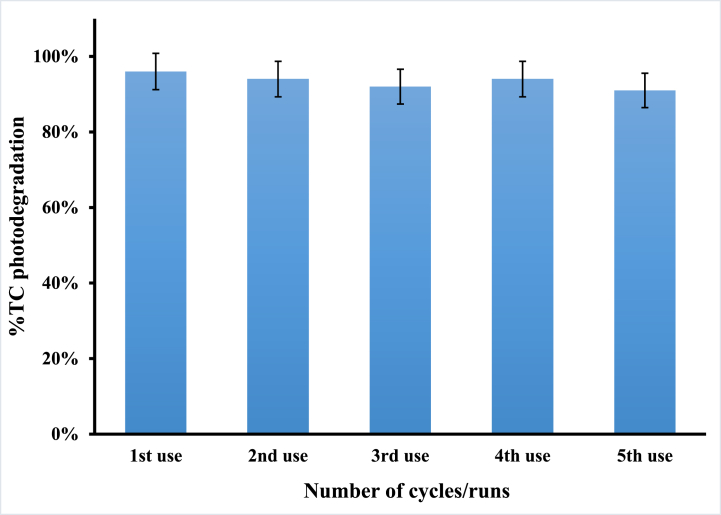
Photocatalytic efficacy retention of CuS/TiO_2_ after recovery and reuse.

Clearly, one of the primary issues in this work was the reusability of the photocatalyst, which had already shown that cycles of photodegradation did not result in losses in the efficiency of the CuS/TiO₂ photocatalyst. The experiments were conducted by using 100 mL of 50 ppm TC and irradiating for 60 min at a pH of 6.1. After each cycle, the catalyst was recovered by filtration, washed with distilled water, and dried for further use in the next cycle. Fig. 10 indicates that the photocatalyst does not exhibit any significant loss in performance, indicating that it is strongly structurally stable and possesses consistent catalytic activity. Therefore, this durability, considering the catalyst, underlines its potential for long-term applications and therefore makes the process cost-effective and sustainable for large-scale wastewater treatment processes. The fact that the photocatalyst can be reused without loss of effectiveness reduces not only the operational costs but also waste, therefore fitting the principles of green chemistry.

More importantly, the use of CuS as a sensitizer provides a less toxic and more secure choice than the extensively used sensitizers CdS and CdSe, which are highly toxic. Owing to its suitable band gap and safety in the environment, CuS functions as a good sensitizer by which the photocatalytic activity of TiO₂ is enhanced without any health or environmental threats. Therefore, these findings highlight the practical advantages of CuS/TiO₂ photocatalysts, in which, with further optimization, this system could support a key technology to address persistent organic pollutants in wastewater by developing the field of environmental remediation. By leveraging some of the unique advantages of using CuS as a sensitizer, this work could provide more efficient and sustainable photocatalytic solutions for environmental cleanup efforts.

## 4 Conclusion

The CuS/TiO₂ photocatalyst shows significant potential for the effective photocatalytic degradation of tetracycline under UV–visible light, making it a valuable tool for environmental remediation. Although CuS/TiO₂ has been previously synthesized, our study highlights its benefits in terms of sustainability and safety compared with more toxic alternatives such as cadmium sulfide and cadmium selenide. CuS acts as a sensitizer, enhancing the photocatalytic process while being environmentally friendly.

The CuS/TiO₂ system achieved high tetracycline (TC) removal efficiencies of up to 95 % at lower concentrations and maintained effective degradation even at higher TC concentrations. The quantum yield (QY) increased with increasing TC concentration, demonstrating efficient photon utilization despite possible active site saturation.

Optimization of the photocatalyst loading showed that the most effective degradation occurred when the amount of CuS/TiO₂ was between 0.15 and 0.20 g, balancing active site availability with photon efficiency. The system also demonstrated consistent reusability, confirming its practicality and sustainability. These results support the use of the CuS/TiO₂ photocatalyst as a feasible solution for wastewater treatment, offering an environmentally friendly method for managing recalcitrant organic pollutants and advancing green technology in environmental cleanup.

## Availability of data and materials

This study does not involve applicable data sharing.

## Ethical approval

This study did not require ethics approval.

## Consent to publish

The author will publicly assume responsibility for the content.

## Declaration of competing interest

I wish to confirm that there are no known conflicts of interest associated with this publication and there has been no significant financial support for this work that could have influenced its outcome.

## References

[1] Qu J., Fan M. (2010). The current state of water quality and technology development for water pollution control in China. Crit. Rev. Environ. Sci. Technol..

[2] Schwarzenbach R.P., Egli T., Hofstetter T.B., Von Gunten U., Wehrli B. (2010). Global water pollution and human health. Annu. Rev. Environ. Resour..

[3] Singh R.L., Singh P.K. (2017).

[4] Ilyas H., Mal J., Masih I., van Hullebusch E.D. (2022). Biotechnology for Environmental Protection.

[5] Rashmi I., Roy T., Kartika K., Pal R., Coumar V., Kala S., Shinoji K. (2020). Contaminants in Agriculture: Sources.

[6] Badran I., Manasrah A.D., Nassar N.N. (2019). A combined experimental and density functional theory study of metformin oxy-cracking for pharmaceutical wastewater treatment. RSC advances.

[7] Lim K.Y., Foo K.Y. (2021). Hazard identification and risk assessment of the organic, inorganic and microbial contaminants in the surface water after the high magnitude of flood event. Environ. Int..

[8] Darwish W.S., Thompson L.A. (2023). Present Knowledge in Food Safety.

[9] Castro-Jiménez C.C., Grueso-Domínguez M.C., Correa-Ochoa M.A., Saldarriaga-Molina J.C., García E.F. (2022). A coagulation process combined with a multi-stage filtration system for drinking water treatment: an alternative for small communities. Water.

[10] Ko Y.G. (2024). Hybrid method integrating adsorption and chemical precipitation of heavy metal ions on polymeric fiber surfaces for highly efficient water purification. Chemosphere.

[11] Oh M., Lee K., Jeon M.K., Foster R.I., Lee C.-H. (2023). Chemical precipitation–based treatment of acidic wastewater generated by chemical decontamination of radioactive concrete. J. Environ. Chem. Eng..

[12] Wu Q., Chen C., Zhang Y., Tang P., Ren X., Shu J., Liu X., Cheng X., Tiraferri A., Liu B. (2023). Safe purification of rural drinking water by biological aerated filter coupled with ultrafiltration. Sci. Total Environ..

[13] Jin L., Sun X., Ren H., Huang H. (2023). Hotspots and trends of biological water treatment based on bibliometric review and patents analysis. Journal of environmental sciences.

[14] Li Y., Dong H., Xiao J., Li L., Chu D., Hou X., Xiang S., Dong Q., Zhang H. (2023). Advanced oxidation processes for water purification using percarbonate: insights into oxidation mechanisms, challenges, and enhancing strategies. J. Hazard Mater..

[15] Jiménez S., Andreozzi M., Micó M.M., Álvarez M.G., Contreras S. (2019). Produced water treatment by advanced oxidation processes. Sci. Total Environ..

[16] Alvarez-Corena J.R., Bergendahl J.A., Hart F.L. (2016). Advanced oxidation of five contaminants in water by UV/TiO2: reaction kinetics and byproducts identification. J. Environ. Manag..

[17] Byrne C., Subramanian G., Pillai S.C. (2018). Recent advances in photocatalysis for environmental applications. J. Environ. Chem. Eng..

[18] Ren H., Koshy P., Chen W.-F., Qi S., Sorrell C.C. (2017). Photocatalytic materials and technologies for air purification. J. Hazard Mater..

[19] Saad I., Ralha N., Abukhadra M.R., Al Zoubi W., Ko Y.G. (2023). Recent advances in photocatalytic oxidation techniques for decontamination of water. Journal of Water Process Engineering.

[20] Teixeira S., Gurke R., Eckert H., Kühn K., Fauler J., Cuniberti G. (2016). Photocatalytic degradation of pharmaceuticals present in conventional treated wastewater by nanoparticle suspensions. J. Environ. Chem. Eng..

[21] Jia J., Liu D., Wang Q., Li H., Ni J., Cui F., Tian J. (2022). Comparative study on bisphenols oxidation via TiO2 photocatalytic activation of peroxymonosulfate: effectiveness, mechanism and pathways. J. Hazard Mater..

[22] Da Silva G.H., Clemente Z., Khan L.U., Coa F., Neto L.L., Carvalho H.W., Castro V.L., Martinez D.S.T., Monteiro R.T. (2018). Toxicity assessment of TiO2-MWCNT nanohybrid material with enhanced photocatalytic activity on Danio rerio (Zebrafish) embryos. Ecotoxicology and environmental safety.

[23] Schneider J., Matsuoka M., Takeuchi M., Zhang J., Horiuchi Y., Anpo M., Bahnemann D.W. (2014). Understanding TiO2 photocatalysis: mechanisms and materials. Chemical reviews.

[24] Guo Q., Zhou C., Ma Z., Yang X. (2019). Fundamentals of TiO2 photocatalysis: concepts, mechanisms, and challenges. Adv. Mater..

[25] Zyoud A.H., Saleh F., Helal M.H., Shawahna R., Hilal H.S. (2018). Anthocyanin‐Sensitized TiO2 nanoparticles for phenazopyridine photodegradation under solar simulated light. J. Nanomater..

[26] Zyoud A., Murtada K., Kwon H., Choi H.-J., Kim T.W., Helal M.H., Faroun M., Bsharat H., Park D., Hilal H.S. (2018). Copper selenide film electrodes prepared by combined electrochemical/chemical bath depositions with high photo-electrochemical conversion efficiency and stability. Solid State Sci..

[27] Zyoud A., Al-Kerm R.S., Al-Kerm R.S., Waseem M., Mohammed H.H., Park D., Campet G., Sabli N., Hilal H.S. (2015). High PEC conversion efficiencies from CuSe film electrodes modified with metalloporphyrin/polyethylene matrices. Electrochim. Acta.

[28] Yu J., Gong C., Wu Z., Wu Y., Xiao W., Su Y., Sun L., Lin C. (2015). Efficient visible light-induced photoelectrocatalytic hydrogen production using CdS sensitized TiO 2 nanorods on TiO 2 nanotube arrays. J. Mater. Chem. A.

[29] Zyoud A.H., Zaatar N., Saadeddin I., Ali C., Park D., Campet G., Hilal H.S. (2010). CdS-sensitized TiO2 in phenazopyridine photo-degradation: catalyst efficiency, stability and feasibility assessment. J. Hazard Mater..

[30] Lo S.-C., Lin C.-F., Wu C.-H., Hsieh P.-H. (2004). Capability of coupled CdSe/TiO2 for photocatalytic degradation of 4-chlorophenol. J. Hazard Mater..

[31] Liu L., Hui J., Su L., Lv J., Wu Y., Irvine J.T. (2014). Uniformly dispersed CdS/CdSe quantum dots co-sensitized TiO2 nanotube arrays with high photocatalytic property under visible light. Mater. Lett..

[32] Li F., Wu J., Qin Q., Li Z., Huang X. (2010). Controllable synthesis, optical and photocatalytic properties of CuS nanomaterials with hierarchical structures. Powder Technol..

[33] Wu H., Li Y., Li Q. (2017). Facile synthesis of CuS nanostructured flowers and their visible light photocatalytic properties. Appl. Phys. A.

[34] Shao Y.-B., Wang L.-H., Huang J.-H. (2016). ZnS/CuS nanotubes for visible light-driven photocatalytic hydrogen generation. RSC advances.

[35] Saranya M., Ramachandran R., Samuel E.J.J., Jeong S.K., Grace A.N. (2015). Enhanced visible light photocatalytic reduction of organic pollutant and electrochemical properties of CuS catalyst. Powder Technol..

[36] Farooq M.H., Aslam I., Shuaib A., Anam H.S., Rizwan M., Kanwal Q. (2019). Band gap engineering for improved photocatalytic performance of CuS/TiO2 composites under solar light irradiation. Bull. Chem. Soc. Ethiop..

[37] Chandra M., Bhunia K., Pradhan D. (2018). Controlled synthesis of CuS/TiO2 heterostructured nanocomposites for enhanced photocatalytic hydrogen generation through water splitting. Inorganic chemistry.

[38] Dai Y., Liu M., Li J., Yang S., Sun Y., Sun Q., Wang W., Lu L., Zhang K., Xu J. (2020). A review on pollution situation and treatment methods of tetracycline in groundwater. Separ. Sci. Technol..

[39] Amangelsin Y., Semenova Y., Dadar M., Aljofan M., Bjørklund G. (2023). The impact of tetracycline pollution on the aquatic environment and removal strategies. Antibiotics.

[40] Saranya M., Santhosh C., Ramachandran R., Kollu P., Saravanan P., Vinoba M., Jeong S.K., Grace A.N. (2014). Hydrothermal growth of CuS nanostructures and its photocatalytic properties. Powder Technol..

[41] Zhou F., Zhang Z., Jiang Y., Yu G., Wang Q., Liu W. (2020). One-step in situ preparation of flexible CuS/TiO2/polyvinylidene fluoride fibers with controlled surface morphology for visible light-driven photocatalysis. J. Phys. Chem. Solid..

[42] Ma J., Du Q., Ge H., Zhang Q. (2019). Fabrication of core–shell TiO2@ CuS nanocomposite via a bifunctional linker-assisted synthesis and its photocatalytic performance. J. Mater. Sci..

[43] Im Y., Kwak B.S., Kang M. (2014). Synthesis of egg-shaped core@ shell structured CuS@ TiO2 particle and its thermal stability. Powder Technol..

[44] Rangel H., Castillo A., Hernández J., Paz J., Montes H., García P., Casillas C., Pérez M., González C.R. (2015). Synthesis of copper sulfide (CuS) thin films by a solid-vapor reaction. Chalcogenide Lett..

[45] Fu Y., Li Q., Liu J., Jiao Y., Hu S., Wang H., Xu S., Jiang B. (2020). In-situ chemical vapor deposition to fabricate Cuprous oxide/copper sulfide core-shell flowers with boosted and stable wide-spectral region photocatalytic performance. J. Colloid Interface Sci..

[46] Mohammed K.A., Ahmed S.M., Mohammed R.Y. (2020). Investigation of structure, optical, and electrical properties of CuS thin films by CBD technique. Crystals.

[47] Zyoud A., AlKerm R.S., Alkerm R.S., Park D., Helal M.H., Campet G., Muthaffar R.W., Kwon H., Hilal H.S. (2016). Enhanced PEC characteristics of pre-annealed CuS film electrodes by metalloporphyrin/polymer matrices. Sol. Energy Mater. Sol. Cell..

[48] Omran A.H., Jaafer M.D. (2013). Annealing effect on the structural and optical properties of CuS thin film prepared by Chemical Bath Deposition (CBD). Journal of Kufa-physics.

[49] Apolinar-Iribe A., Acosta-Enriquez M., Berman-Mendoza D., Mendivil-Reynoso T., Larios-Rodriguez E., Ramirez-Bon R., Castillo S. (2013). Effects of the annealing on CUS thin films using triethanolamine as complexing agent by CBD. Chalcogenide Lett..

[50] Agoro, A.M., INTEGRATION OF NANOSTRUCTURED METAL SULFIDES INTO TITANIUM (IV) OXIDE FOR HIGH PERFORMANCE DYE SENSITIZED SOLAR CELL.

[51] Zyoud A.H., Asaad S., Zyoud S.H., Zyoud S.H., Helal M.H., Qamhieh N., Hajamohideen A., Hilal H.S. (2020). Raw clay supported ZnO nanoparticles in photodegradation of 2-chlorophenol under direct solar radiations. J. Environ. Chem. Eng..

[52] Zyoud A.H., Zubi A., Hejjawi S., Zyoud S.H., Helal M.H., Zyoud S.H., Qamhieh N., Hajamohideen A., Hilal H.S. (2020). Removal of acetaminophen from water by simulated solar light photodegradation with ZnO and TiO2 nanoparticles: catalytic efficiency assessment for future prospects. J. Environ. Chem. Eng..

[53] Zyoud A.H., Hilal H.S. (2009). Silica-supported CdS-sensitized TiO2 particles in photo-driven water purification: assessment of efficiency, stability and recovery future perspectives. Chapter in a book, Water Purification, Novascience Pub, NY (in Press, 2008).

